# Renal Damaging Effect Elicited by Bicalutamide Therapy Uncovered Multiple Action Mechanisms As Evidenced by the Cell Model

**DOI:** 10.1038/s41598-019-39533-3

**Published:** 2019-03-04

**Authors:** Chiung Chi Peng, Chang-Yu Chen, Chang-Rong Chen, Chang-Jui Chen, Kun-Hung Shen, Kuan-Chou Chen, Robert Y. Peng

**Affiliations:** 1 Graduate Institute of Clinical Medicine, School of Medicine, College of Medicine, Taipei Medical University, 250 Wu-Hsing Street, Taipei, 11031 Taiwan; 2 Wayland Academy, 101 North University Avenue, Beaver Dam, WI 53916 USA; 3 International Medical Doctor Program, The Vita-Salute San Raffaele University, Via Olgettina 58, 20132 Milano, Italy; 4 Division of Urology, Department of Surgery, Chi Mei Medical Center, Tainan, 710 Taiwan; 5 Department of Optometry, College of Medicine and Life Science, Chung Hwa University of Medical Technology, Tainan, 717 Taiwan; 6 Department of Urology, Taipei Medical University Shuang-Ho Hospital, 291, Zhong-Zheng Rd., Zhong-He, Taipei, 23561 Taiwan; 7 Department of Urology, School of Medicine, College of Medicine, Taipei Medical University, 250 Wu-Shing St., Taipei, 11031 Taiwan; 8 Research Institute of Biotechnology, School of Medicine and Nursing, Hungkuang University, No.1018, Sec. 6, Taiwan Boulevard, Shalu District, Taichung City, 43302 Taiwan

## Abstract

Bicalutamide (Bic) is frequently used in androgen deprivation therapy (ADT) for treating prostate cancer. ADT-induced hypogonadism was reported to have the potential to lead to acute kidney injury (AKI). ADT was also shown to induce bladder fibrosis via induction of the transforming growth factor (TGF)-β level. We hypothesized that Bic can likely induce renal fibrosis. To understand this, a cell model was used to explore expressions of relevant profibrotic proteins. Results indicated that Bic initiated multiple apoptotic and fibrotic pathways, including androgen deprivation, downregulation of the androgen receptor → phosphatidylinositol-3-kinase → Akt pathway, upregulation of the extrinsic apoptotic pathway- tumor necrosis factor α →  nuclear factor κB → caspase-3, increased expressions of fibrosis-related proteins including platelet-derived growth factor β, fibronectin and collagen IV, and enhanced cell migration. The endoplasmic reticular stress pathway and smooth muscle actin were unaffected by Bic. Co-treatment with testosterone was shown to have an anti-apoptotic effect against Bic, suggesting a better outcome of Bic therapy if administered with an appropriate testosterone intervention. However, since Bic was found to inhibit the membrane transport and consumption rates of testosterone, a slightly larger dose of testosterone is recommended. In conclusion, these pathways can be considered to be pharmaceutically relevant targets for drug development in treating the adverse effects of Bic.

## Introduction

Chronic kidney disease (CKD) has become a major worldwide health issue that has attracted much attention. Renal fibrosis (RF) with diverse etiologies is usually considered to be a common pathological hallmark of many advanced kidney diseases. Clinically, RF is the most reliable predictor reflecting the progression from CKD to end-stage renal failure (ESRD)^1^.

Accumulating evidence now indicates that renal inflammation plays a key role in progressive kidney disease^2^. Renal inflammatory and fibrotic signaling commonly involved in CKD is thought to involve nuclear factor (NF)-κB, tumor necrosis factor (TNF)-α, and platelet-derived growth factor (PDGF)^2,3^. Almost all human renal diseases are characteristically related to some altered expression of PDGF components^3^.

Bicalutamide (Bic, Casodex) is a nonsteroidal pure antiandrogen (an androgen receptor (AR) antagonist)^4^. Currently, Bic has become the most widely prescribed antiandrogenic medicine for treating prostate cancer (PCa)^5^, usually given as monotherapy (150 mg once daily) for treating early nonmetastatic PCa^6^. Generally, Bic (50 mg, u.i.d) is administered as combined therapy with a luteinizing hormone-releasing hormone (LHRH) agonist or surgical castration for treating advanced PCa^6^. Androgen-deprivation therapy (ADT)-induced hypogonadism was reported to have the potential to lead to acute kidney injury (AKI)^7^. Up to 36.67% of people who have taken Bic therapy for 1~6 months may experience kidney failure^8^. An interdisciplinary study tried to explain the effect of reduced testosterone levels, which might be relevantly associated with the renal-damaging effect of Bic^9^. Testosterone appears to protect the kidneys by improving blood flow. Bic blocks the body’s ability to use androgens. Reducing the serum androgen concentration may damage the tiny capillaries that filter wastes from the blood stream, thereby triggering AKI^9^.

Bic disrupted of telomeric complexes in androgen receptor(AR)-positive LNCaP cells, but had less of an effect in AR-negative PC-3 cells^10,11^. Maintaining the integrity and length of telomeres is essential for genomic stability, and normal growth and survival of mammalian cells^12^. A short telomere length was associated with CKD progression among smokers (*p* = 0.001) and diabetic patients (*p* = 0.03). Shortening of the telomere length might be associated with CKD prevalence/occurrence or declining kidney function^13^.

Recently, Bic therapy was documented to induce lung disease^14^, liver injury^15^, and hepatotoxicity^16^. Those cases raised practical warnings to clinical physician that, while rare, clinically significant and potentially life-threatening side effects can result from the use of Bic.

The AR plays a key role in regulating of gene expressions in tissues like the prostate and kidneys^17–19^, in particular, it plays a critical role in castration-resistant PCa (CRPC)^19^. A vast network of signaling pathways is more or less deregulated in PCa, resulting in PCa progression^20^. ADT was reported to induce a diversity of modalities of apoptosis, autophagy, necroptosis, and necrosis^21^.

Critical AR-mediated or AR-associated signaling pathways are closely linked to the phosphatidylinositol-3-kinase (PI3K)/Akt pathway, receptor tyrosine kinases, the p38 mitogen-activated protein kinase (MAPK) pathway, and the Wnt/β-catenin pathway^21^. Among these, the most relevant is the PI3K-Akt pathway^21^. Activation of Akt usually acts as an important mode associated with a variety of signaling cascades that are relevantly linked to kidney damage and RF^22^.

CKD usually exhibits several prominent morphologic features including infiltration by monocytes and/or macrophages, RF, and loss of native renal cells^23^. Progressive kidney diseases also manifest by apoptosis in glomeruli and tubules^24,25^.

It seems that a higher percentage of males suffer from kidney failure than females when taking Bic (eHealthMe personalized health information)^8^. To the present, a linking between Bic therapy and RF is still lacking. In a preliminary experiment with a diabetic rat model, we found that the severity score of diabetic nephropathy in diabetes mellitus (DM) rats was only 2.0, while the combination of DM and Bic enhanced the score to 3.0. This indeed inspired our hypothesis that Bic, under some pathologically favorable circumstances, could induce renal damage, eventually leading to RF.

To understand this, an *in vitro* RMC cell/high-glucose medium model was carried out to elucidate the relevant molecular mechanism associated with renal damage and/or RF induced by Bic and intervention with testosterone as a protective co-therapy. To our knowledge, this is the first report to adopt a cell model to examine the possible role of Bic in inducing RF.

## Materials and Methods

### Chemicals

Bicalutamide, testosterone, R1881 (methyltrienolone, a synthetic androgen), etoposide, acetonitrile, formic acid, ammonia, 3-(4, 5-dimethylthiazol-2-yl)-2, 5-diphenyltetrazolium bromide (MTT) and TEMED (tetramethylethylene diamine) were provided by Sigma-Aldrich (St. Louis, MO, USA). The enhanced chemiluminescence (ECL) system was a product of Merck Millipore. (Billerica, MA, USA). The PRO-PREP Protein Extraction Solution was provided by iNtRON Biotech. (Kyungki-Do, Korea). Human recombinant TGF-β1 was provided by BioVision. (Milpitas, CA, USA). The semi-quantitative total tissue collagen detection kit (Sirius Red/Fast Green collagen staining kit) was provided by Chondrex, Inc. (Redmond, WA, Australia). The Akt activator SC79 and inhibitor MK-2206 were provided by Selleck Chemicals (Houston, TX, USA). All other chemicals were purchased from Wako Pure Chemicals (Osaka, Japan) unless otherwise stated.

### Source of cell lines

NRK52E, a rat normal renal proximal tubular epithelial cell line; and RMC, the rat mesangial cell line, were provided by the Bioresource Collection & Research Center (BCRC; Hsinchu, Taiwan).

### Cell cultures and MTT assay

The RMC cell line was incubated in modified Dulbecco’s Eagle’s medium (DMEM) containing 4 mM L-glutamine, 1.5 g/L sodium bicarbonate, 4.5 g/L glucose and supplemented with 0.4 mg/mL G418, 15% fetal bovine serum, and 0.4% PBS. NRK52E cells were cultured in DMEM containing 2 mM glutamine, 1% non-essential amino acids, 5% fetal bovine serum (FBS) and 0.4% PBS.

The cells were inoculated onto 24-well plates at a density 1.5 × 10^4^ cells/well (RMC) or 3.5 × 10^4^ cells/well (NRK52E), gently agitated to mix them well, and incubated overnight until completely adherent. The medium was removed and replaced with medicine (either testosterone or bicalutamide) containing 2% charcoal-treated FBS medium, and treated for 24 and 48 h. Doses of testosterone used were 1, 10, 100, and 1000 nM, while those of Bic was 3.75, 7.5, 15, 30, and 60 μM. After cells were challenged with drugs and incubated for 24 and/or 48 h, cell viability was measured with MTT and formazan production was determined by the absorbance at 540 nm with a multifunctional plate reader (Bio-Rad, CA, USA). In cases where SC-79 (an Akt activator) and MK-2206 (an Akt inhibitor) were applied, cells were pretreated with these modulators, 10 μM of SC-79 and/or 1 μM of MK-2206, for 30 min before the addition of Bic.

### Transwell assay for RMC cells

The transwell method was carried out as previously reported by Peng *et al*.^26^. The dose of Bic was 30 μM (which produced a viability around 50%) in parallel with TGF-β at a dose of 5 ng/ml as the positive control. In brief, 1 day before the experiment, a sufficient number of cells was inoculated in DMEM containing 2% charcoal-treated FBS on a 24-well plate with inserts. A density of 10^5^ cells /mL was seeded in the insert and incubated in the incubator for 72 h until cells were adherent. When the set time was reached, the inserts were taken out, medium remaining inside was removed, and inserts were blotted with a PBS-wetted cotton plug. Inserts were immersed in 10% formalin for at least 20 min and then rinsed with PBS. The treated inserts were dipped in an eosin solution for at least 30 min and rinsed several times with double-distilled water (ddH_2_O). Inserts were immersed in a hematoxylin solution for at least 30 min and then rinsed several times with ddH2O. Micrographs were observed with a light microscope at a magnification ×200. Five-fields examined in each group. The number of transwell cells was counted from photos. The experiment was performed in triplicate.

### Quantification by liquid chromatographic (LC)/tandem mass spectrometric (MS/MS) analysis

#### Preparation of calibration curve

Authentic testosterone and Bic at 1 mg each, were exactly weighed and dissolved in 1 mL acetonitrile to make stock solutions (1 mg/mL or 1000 ppm). The testosterone stock solution was diluted with acetonitrile to concentrations 0.0125, 0.025, 0.05, 0.10, and 0.20 ppm. These diluted standard solutions were used for calibration. Acetonitrile was used as the blank. The stock solution of Bic was prepared by dissolving 1 mg of Bic in 1 mL of acetonitrile to make the stock solution with a concentration of 1 mg/mL. This was diluted with acetonitrile to concentrations of 3.125, 6.25, 12.5, and 25 μg/mL, to establish the calibration curve.

#### Preparation of samples from culture media

Sample media (1 mL of each) were extracted with acetonitrile (1 mL) at a volume ratio of 1:1) and vortexed for 5 min. Sample tubes were agitated on a shaker with the bottom of the tubes immersed in an ice bath for 10 min, then centrifuged at 4 °C, and 15000 × g for 10 min. The extraction was repeated three times, with 1 mL of fresh acetonitrile being replaced each time. The supernatants were collected in a 2-mL fresh tubes and stored at −20 °C. One to two days prior to being subjected to the LC/MS/MS analysis, the refrigerated samples were removed and lyophilized. Desiccated samples were redissolved in fresh acetonitrile.

#### LC/MS/MS analysis

After being redissolved in acetonitrile, samples were immediately sent to the Core Facility at Taipei Medical University after redissolved with aetonitrile. A Thermo XSERIES 2 ICP-MS (Thermo Scientific, Waltham, MA, USA) was attached with a C18 column. The mobile phase used for analysis of testosterone consisted of 70% acetonitrile (containing 0.1% formic acid in ddH_2_O), while that for Bic consisted of 70% acetonitrile (containing 0.1% ammonia in ddH_2_O). The mobile phase solution was agitated to drive off gas bubbles being subjected to LC/MS. Authentic testosterone and Bic were similarly treated and subjected to analysis to establish calibration curves from which the extracellular concentration of testosterone was calculated. The detection limit was 0.01 ∼ 1 ppm (i.e. 10 ng/mL~1 μg/mL).

### Flow Cytometric Analysis

The protocol of the cell cycle analysis was followed instructions of Cell Signaling Technology (Danvers, MA, USA). Cells were collected by centrifugation and resuspended in 1 mL 1x PBS. Formaldehyde was added to obtain a final concentration of 4% formalin. Cells fixed for 10 min at 37 °C and chilled on ice for 1 min. Fixed cells were removed by centrifugation for 10 min at 5000 × g, resuspended in 90% ethanol as described above, incubated for 30 min on ice. The cells were counted using a hemocytometer. An aliquot of 10^6^ cells was transferred into each assay tube (by volume). Cells were washed with 2~3 mL of incubation buffer, centrifuged, and resuspended in 0.5 mL of DNA dye (propidium iodide (PI)/RNase Staining Solution #4087), incubated for 30 min at ambient temperature and analyzed in a DNA-staining solution with a flow cytometer. The Muse Annexin V & Dead Cell Assay was carried out for the quantitative analysis of live cells, early and late apoptosis, and cell death on both adherent and suspended cell lines on a Muse Cell Analyzer (MCH100105 Muse® Annexin V and Dead Cell Assay Kit) as instructed by the supplier (Merck-Millipore, Dresden, Germany).

### Protein Extraction

#### Cytosolic protein extraction

To lysis buffer (10 mL), 100 μL of protease inhibitor was added and mixed well to create protein inhibitor lysis buffer (PILB). One hundred milligrams of experimental cells was mixed with 5 mL of PILB, 1 mL of protein extraction solution (EDTA-free) (Intron Biotechnology, MA, USA) was added, and cells were homogenized. The homogenate was incubated on ice for 40 min and centrifuged at 4 °C and 14,000 × g for 20 min. The supernatant was transferred to a new microcentrifuge tube and stored at −80 °C for future use.

#### Nuclear protein extraction

Residual cell pellets were used for extraction of nuclear proteins according to the manufacturer’s instructions (cat. no. 786–182., G-Biosciences, St. Louis, MO, USA).

### Western Blot Analysis

The lysate containing an approximate amount (50 mg) of protein was boiled in PBS for 10 min. The sample solutions, after being cooled, were loaded onto a 7.5% polyacrylamide sodium dodecylsulfate (SDS) gel. Proteins were transferred to a polyvinylidene difluoride (PVDF) membrane and then rinsed with TBS Tween buffer (TBST) and blocked at 4 °C overnight in TBST containing 5% w/v non-fat milk. The primary antibodies used were TNF-α, Bax, histone (GeneTex, Irvine, CA, USA), nuclear factor (NF)-κB, CHOP (Epitomics, Burlingame, CA, USA), Fas, collagen IV, β-actin (Novus Biologicals, Littleton, CO, USA), calpain-1, caspase-12 (Abcam, Cambridge, MA, USA), caspase-3, Akt, phosphorylated (p)-Akt, PDGF receptor (PDGFR)-β, p-PDGFR-β, PI3K, p-PI3K, Bcl-2, TNF-receptor (Cell Signaling, Danvers, MA, USA), androgen receptor (AR), p-PI3K, PI3K, fibronectin (Santa Cruz Biotechnology, Santa Cruz, CA, USA), and smooth muscle actin (SMA) (Sigma-Aldrich, St. Louis, MO, USA) in TBST. The PVDF membrane was then rinsed with TBST and incubated with secondary antibodies for 1 h at room temperature. Expressions of proteins were detected with Immobilon Western horseradish peroxidase (HRP) Substrate (Millipore, Burlington, MA, USA). β-Actin or histone was used as the reference protein. The experiments were performed in triplicate.

### Statistical Analysis

Statistical analyses were performed by Student’s *t*-test with the SPSS 10.0 computer statistical software (SPSS, Chicago, IL, USA). An analysis of variance (ANOVA) was also used with Tukey’s test to analyze variances and significances of difference between paired means. The significance of the difference was judged by confidence levels of *p < 0.05, **p < 0.01, ***p < 0.005, and ^#^*p* < 0.05, ^##^*p* < 0.01, ^###^*p* < 0.005.

## Results and Discussions

### Bic decreases the cell viability of renal mesangial cells, but not that of renal tubule cells

**Figure 1:**
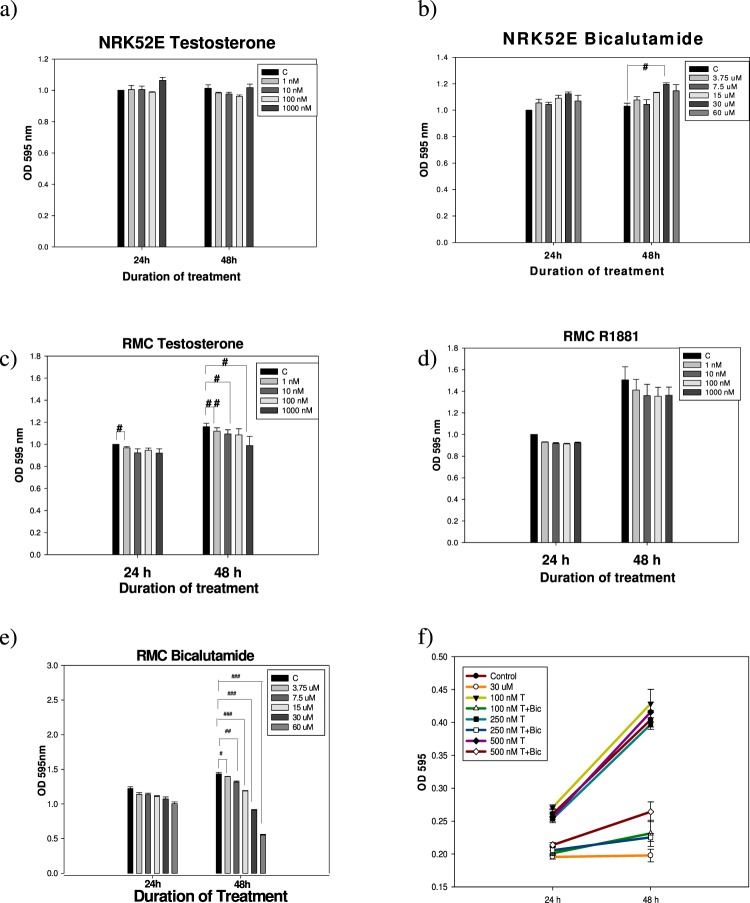
MTT assay of NRK52E- and RMC cells affected by testosterone, bicalutamide, and R1881 in 25 mM hyperglycemic medium. NRK52E cell line (1 × 10^4^ cells/well) on 24-well plate was incubated in medium containing 5% bovine serum and 0.4% PBS overnight until adhered. Finally, the medium was replaced fresh with serum free medium. RMC cell line (1 × 10^4^ cells/well) on 24-well plate was incubated in medium containing 25 mM glucose, 15% FBS, 0.4% PBS, and 0.8% G418 overnight until adhered. Finally, the medium was replaced fresh with 2% FBS. At final step, the two cells were simultaneously treated as indicated. (**a**) NRK52E + testosterone (1~10^3^ nM), (**b**) NRK52E + bicalutamide (3.75~60 μM), (**c**) RMC + testosterone (1~10^3^ nM), (**d**) RMC + R1881 (1~10^3^ nM), and (**e**) RMC + bicalutamide, and (**f**) RMC + bicalutamide + testosterone for duration as indicated. Experiment was performed in triplicate

The proliferative behavior of NRK52E cells seemed to be totally unaffected by treatment with testosterone (Fig. 1a), in contrast, Bic slightly increased their growth in a time- and dose-dependent manner (Fig. 1b). Conversely, testosterone showed an apparent time-dependent growth-promoting- yet a dose-dependent suppressive effect on RMC cells in 25 mM glucose medium (Fig. 1c). A similar effect was also seen for R1881 (methyltrienolone, an AR agonist) (Fig. 1d). Bic (3.75~60 μM) acted in the same manner but more prominently (Fig. 1e).

This phenomenon (Fig. 1a) was very interesting and worth extensive exploration. As is well known, both the NRK52E and RMC cell lines exhibit AR expression^27–29^, while AR expression in mouse proximal tubular cells (like mProx24) is much greater than that in mouse mesangial cells (e.g., MES13)^30^. So why was the growth of NRK52E cells totally independent of testosterone (Fig. 1a)? We speculated that this could have been due to the following reasons: (1) The affinity of testosterone for the AR in NRK52E cells may be comparatively lower than that in RMC cells; and (2) The main function of the AR in NRK52E cells may be biased toward being associated with other physiological roles. The literature demonstrates that NRK52E cells are directly associated with testosterone-induced senescence^28^, while expression of the renal senescence marker protein, SMP30, was upregulated by endogenous testosterone stimulation; the latter increases renal anti-aging klotho gene expression via an AR-mediated pathway^29^.

When treated with free testosterone at 100~500 nM, normal proliferation of RMC cells was achieved after 48 h (Fig. 1f). Combined therapy of testosterone and Bic (30 μM) severely retarded their proliferation (Fig. 1f), implying that the antiandrogenic Bic was able to affect the growth of renal mesangial cells via the AR.

AR expression and function were found in micro-dissected glomeruli and cultured mesangial cells^27^. As reported, Bic caused dramatic disruption of telomeric complexes and cell death^10,11^. Alternately, it is worth noting that CKD can be sex-specific or sex-differentiated. Sex hormones may influence a diversity of cellular processes implicated in the pathogenesis of renal disease progression^31^. Sex-specific profibrotic and proinflammatory phenotypes were reported to underlie chronic renal diseases in rat mesangial cells^32^, suggesting that male mesangial cells inherently exhibit a greater risk of renal fibrotic pathogenesis, i.e., chronic renal disease progresses more rapidly in males than in females^32^.

One problem possibly may be raised curious readers would be “Does androgen deprivation-induced CKD directly reflect the status of CKD occurring among females?” Pathologically, female CKD can be induced by many etiological factors that are common to those found in males; however, as for the male CKD caused by androgen-deprivation, like Bic therapy, the situation turns out to be quite different between the two genders. Clearly, antiandrogen is not equal to estrogenic. More importantly, females have less opportunity to receive ADT. Hence the pathological symptoms are not comparable.

### LC/MS/MS analysis of testosterone in culture medium

**Figure 2:**
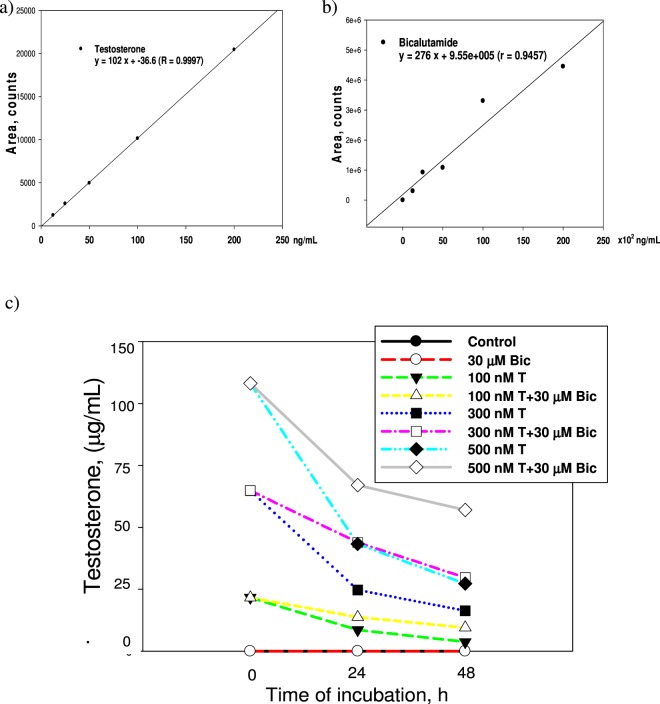
LC/MS/MS analysis of testosterone. (**a**) The standard curve of testosterone. (**b**) The standard curve of bicalutamide. (**c**) The time–dependent change of extracellular concentration of testosterone.

Figure 2 shows the LC/MS/MS data obtained from different drug treatments. No trace of testosterone was detected in the Bic (30 μM) control (Fig. 2). Attractively, when testosterone was co-treated with Bic (30 μM), the overall consumption rate (this is the combined transport rate from the extracellular into the intracellular compartment and the intracellular consumption rate) of testosterone was found to be retarded by the coexisting Bic. For free testosterone at 500, 300, and 100 nM, overall consumption rates were 4.175, 2.982, and 0.785 μg/mL-h, respectively. When testosterone was co-treated with Bic (30 μM), corresponding values changed to 3.300, 1.773, and 0.493 μg/mL-h, respectively, obviously implying a prominent suppressive effect exhibited by Bic against the overall consumption rate of coexisting testosterone (Fig. 2). It is suggested that such competition might have occurred on the AR or the membrane transport gate.

To our best knowledge, this is the first report indicating that pharmacodynamically Bic not only acts as an antagonist against testosterone, but pharmacokinetically can also retard the overall consumption rate of testosterone.

### RMC cell cycle populations were dose- and time-dependently affected by Bic

**Figure 3:**
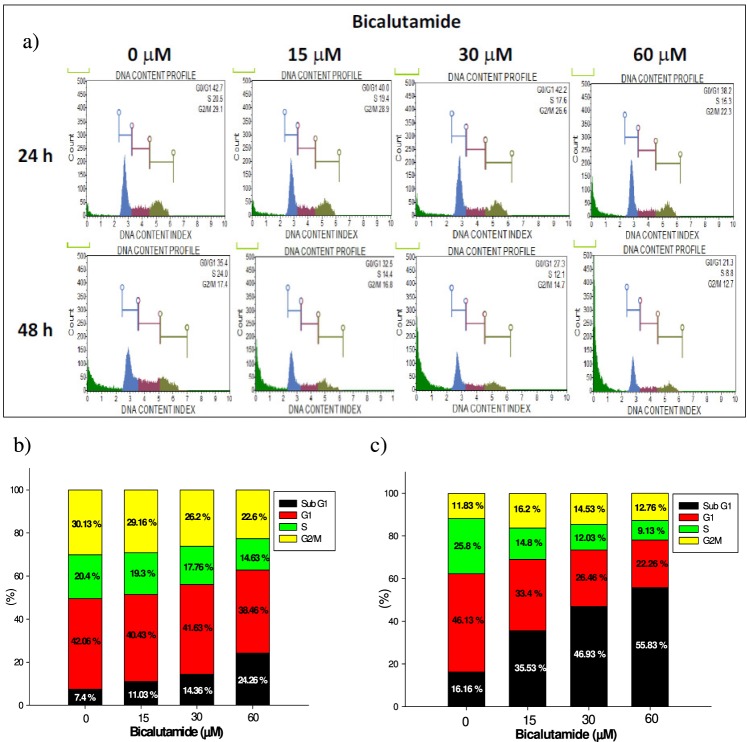
The cell cycle analysis of RMC cell line affected by bicalutamide treatment. (**a**) Histogram for flowcytometric analysis. (**b**) Quantification of cell populations in 24 h. (**c**) Quantification of cell populations in 48 h. Experiment was performed in triplicate

Taking the DNA content and intensity of the fluorescence as a measure of the cell population in each cell cycle phase (Fig. 3a), we found that subG_1_ and G_0_/G_1_ apoptosis of RMC cells occurred in dose- and time-dependent manners (Fig. 3a–c). The subG_1_ population markedly increased from 7.4% (control) to 24.26% (60 μM Bic) within the first 24 h (Fig. 3b) and progressed to 55.83% when treated with 60 μM Bic for 48 h (Fig. 3c). At the same time, the S phase population was found to have drastically declined in a dose- and time-dependent manner (Fig. 3b,c), implying a decrease in the cell population after DNA replication had finished. Such an outcome could have been caused by a dramatic disruption of telomeric complexes by Bic therapy, as cited elsewhere in the literature^10,11^.

The literature also indicates that Bic-induced cytotoxic and cytostatic effects in androgen-positive PCa LNCaP cells could be associated with a marked G_1_ phase arrest and S phase depression^33^. Consistent with this, the same mechanism applied similarly to RMC cells (Fig. 3a–c).

### Bic induces late apoptosis of RMC cells

**Figure 4:**
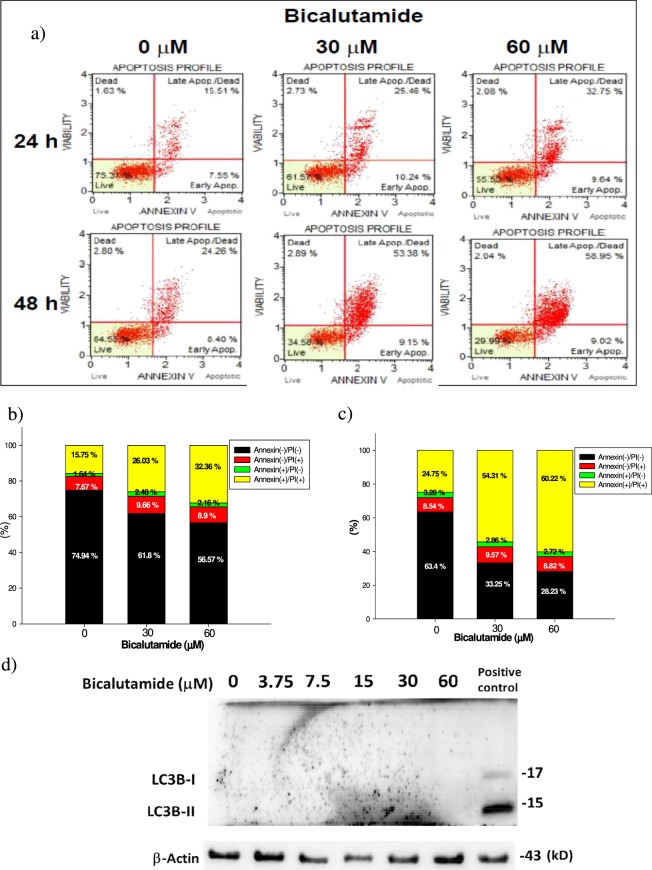
Late apoptosis of RMC cell lines affected by treatment of bicalutamide. (**a**) The apoptosis profile affected by treating with bicalutamide respectively at 24 and 48 h when examined with Annexin V and 7-ADD staining. (**b**) The quantified cell population in each quadrant of (**a**) at 24 h. (**c**) The quantified cell population in each quadrant of (**a**) at 48 h. (**d**) The representative protein expression of autophagic marker LC3B-I and LC3B-II after Bic treatment for 48 h. Late apoptosis of RMC cell lines affected by bicalutamide. Experiment was performed in triplicate (n = 3).

When viewed with combined Annexin V and 7-AAD staining, late apoptosis was seen to have occurred after treatment with Bic (Fig. 4a–c). The apoptotic population increased in a dose- and time-dependent manner. After 24 h of treatment, the late apoptotic population had increased from 15.75% in the control to 26.03% and 32.36% at 30 and 60 μM, respectively. In contrast, after being treated for 48 h, the corresponding values increased from 24.26% for the control to 53.36% and 58.95%, respectively, while no significant change was seen for early apoptosis (Fig. 4a), apparently supporting the occurrence of late apoptosis. Bic was implicated as an underlying cause of cancer cell apoptosis^34,35^. This is believed to be the first report about late apoptosis elicited by Bic in mesangial cells. On the other hand, Bic showed no apparent suppressive effect on the viability of NRK52E cells (Fig. 1b). We wondered “could such a phenomenon as apoptosis be relevantly associated with an alternate cell protective effective that has always been called ‘autophagy’? Results seemed to make this unlikely, because that was no trace of LC3-phosphatidylethanolamine conjugate-II (LC3-II) detected at 48 h (Fig. 4d)^36^. Contrasting this phenomenon associated with NRK52E cells, Bic seemed to play a role of mainly inducing apoptosis of RMC cells. Speculating that such an apoptotic effect might be associated with the caspase signaling pathway, we performed the following study.

### The TNF-α → NF-κB → caspase-3 apoptotic pathway is involved

**Figure 5:**
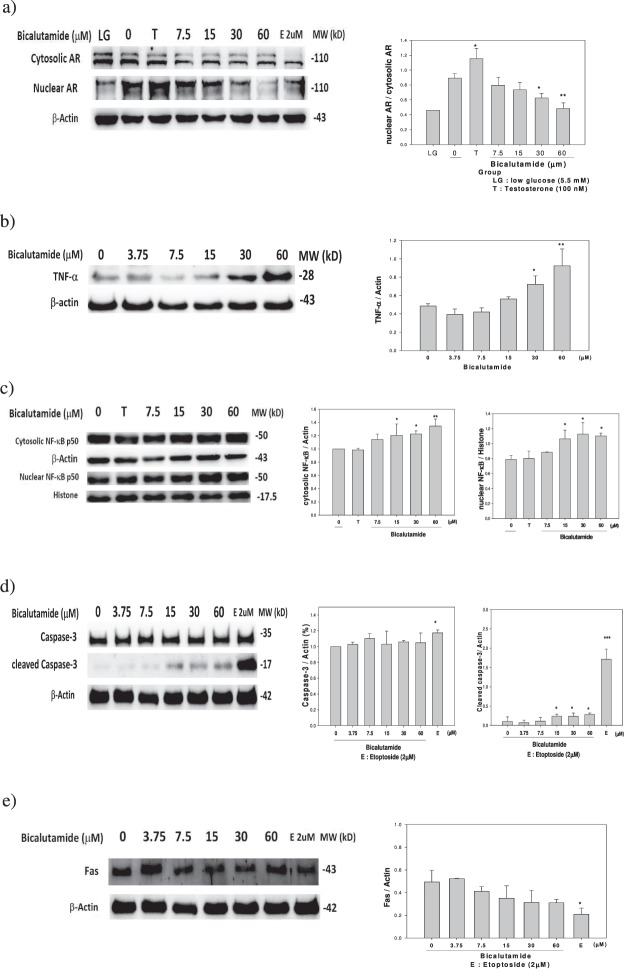
The representative expressions and the quantifications of proteins regarding the bicalutamide-induced apoptosis. (**a**) downregulation of nuclear androgen receptor (nAR), upregulation of (**b**) TNF-α, (**c**) cytosolic and nuclear NFκB p50, (**d**) pro- and cleaved- caspase 3, but not via (**e**) Fas. Experiment was performed in triplicate (n = 3). LG: low glucose 5.5 mM; T: Testosterone 100 nM.

Bic at dose ≥30 μM dose-dependently downregulated the nuclear AR (p < 0.05) (Fig. 5a) and upregulated cytosolic TNF-α (*p* < 0.05) (Fig. 5b). Bic also dose-dependently upregulated the cytosolic and the nuclear NF-κB protein expression (at doses ≥15 μM, *p* < 0.05) (Fig. 5c). While the caspase-3 seemed to remain unchanged, the cleaved caspase-3 was significantly upregulated by Bic at dose ≥15 μM (*p* < 0.05) (Fig. 5d). In contrast to this, Fas expression was totally unaffected (Fig. 5e).

TNF-α induces apoptosis and activates the NF-κB transcription factor^37^. TNF-α and IL-1α were reported to induce apoptosis in mesangial cells^38,39^. Overexpression of IκB may potentiate TNF-α-induced apoptosis and augment caspase-8 and caspase-3 activities in mesangial cells^40^.

NF-κB plays a crucial role in immune and inflammatory responses and protects cells from apoptosis^41^. Upregulation of NF-κB implies that NF-κB is involved in proapoptotic activity as previously demonstrated by Stark *et al*.^42^. Taking these results together (Fig. 5b,c), it can be assumed that the apoptotic pathway might consist of TNF-α → (death domain) → NF-κB → expression of survival genes as often mentioned^43^. Defects in this extrinsic pathway are linked to several disease states, including cancer and kidney diseases^44,45^.

It is suggested that the phenomenon of the upregulation of NF-κB (Fig. 5c) by Bic might be associated with its role as a survival savior. NF-κB activity is essential for cellular homeostasis. Taken together, part of the apoptotic mechanism exerted by Bic may likely involve the extrinsic TNF-α → NF-κB → caspase-3 apoptotic pathway.

### Are the intrinsic and ER stress pathways involved in the process of renal damage?

**Figure 6:**
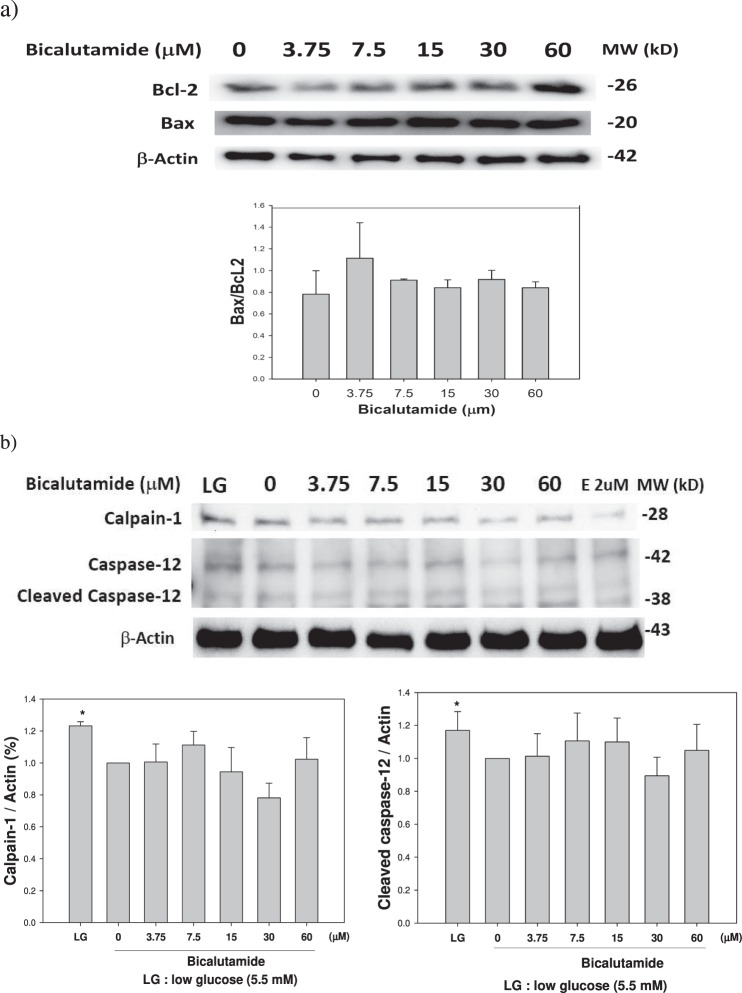
Effect of bicalutamide on expression of proteins related with the intrinsic and ER stress pathways. (**a**) The intrinsic pathway related proteins Bcl-2 and Bax. (**b**) The ER–associated calpain-1 and caspase-12 proteins. Western blots were performed in triplicate and quantified (n = 3). LG: low glucose 5.5 mM; E: Etoptoside 2 μM.

Suspecting the possibility that the intrinsic pathway might be involved in Bic-induced RF^46,47^, we examined expressions of apoptotic (Bax) and antiapoptotic (Bcl-2) proteins, and ER stress-related calpain-1 and caspase-12. Bic at a dose ≥15 μM seemed to have slightly upregulated the Bcl-2 and Bax protein (Fig. 6a). As a consequence, the Bax/Bcl-2 ratio remained unchanged (Fig. 6a). On the other hand, calpain-1, caspase-12 and cleaved caspase-12 proteins were entirely unaffected (Fig. 6b), implying that they obviously have no involvement in the intrinsic and ER stress pathways.

### Could the PI3K-Akt pathway be associated with renal cell apoptosis induced by Bic?

**Figure 7:**
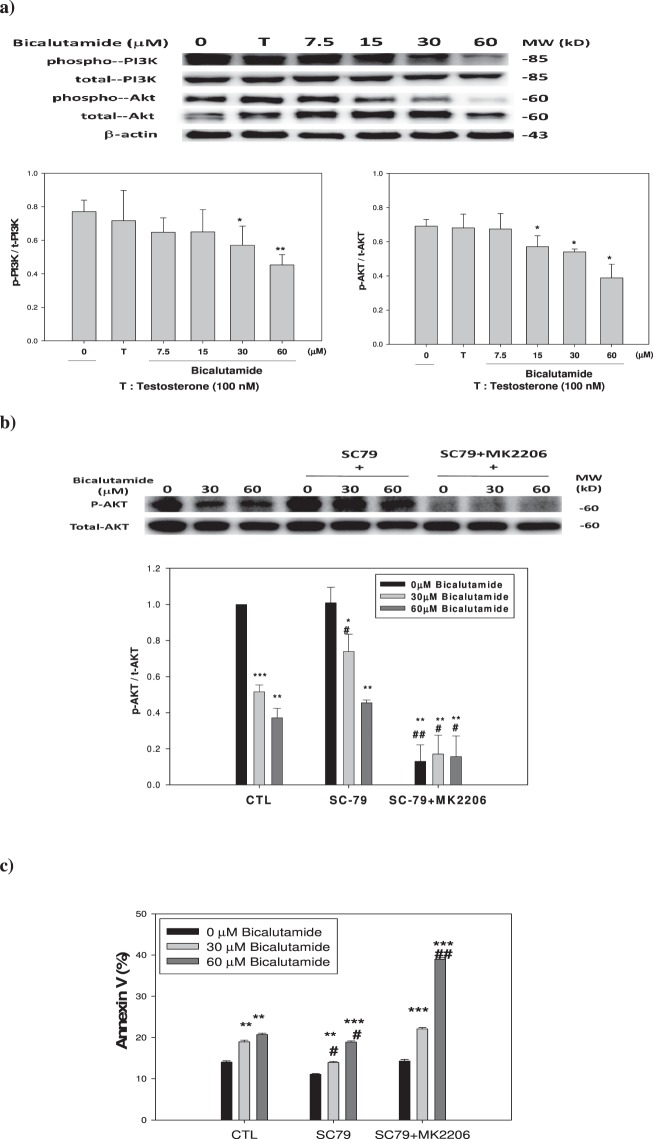
Expression of signaling proteins and Annexin V% affected by testosterone and bicalutamide. (**a**) PI3K and Akt proteins. (**b**) The expression of p-Akt and total Akt when treated as indicated. (**c**) Apoptotic effect of SC79 and a combination of SC79 plus MK2206. The dose(s) used were: testosterone (T) 100 nM; bicalutamide (7.5, 15, 30 and 60 μM); SC79 10 μM, and MK2206 1 μM. Triplicate experiments were statistically treated. Data expressed in mean ± S.D. (n = 3).

As mentioned above, RMC cells responded to Bic *via* caspase-3 dependency. To determine whether there was any further evidence of an intervention by upstream cell survival signaling, we surveyed the PI3K-Akt pathway. PIP3 activates AKT *via* phosphorylation to form p-Akt which in turn activates a diversity of molecules involved in cell survival and proliferation^48^. As seen, the ratio p-PI3K/t-PI3K was downregulated by Bic at dose 30 μM (p < 0.05) and at 60 μM (p < 0.01), and at the same time, accompanied by downregulation of p-Akt (Fig. 7a) by Bic at doses ≥15 μM (p < 0.05). To further understand and confirm this, we used SC-79 (an Akt activator) and MK-2206 (an Akt inhibitor). As can be seen in Western blots of p-Akt and total Akt, results indicated that p-Akt expression was dose-dependently inhibited by Bic, and the Akt activator, SC79, effectively attenuated such suppression, while the combination of SC79 and MK2206 strongly abolished restoration of p-Akt (Fig. 7b). Correspondingly, an apoptotic response was found in the Annexin V assay. In the controls, percentages of Annexin V appeared in a dose-dependent fashion from 14.02% ± 0.32% to 18.95% ± 0.40% and 20.68% ± 0.40% with Bic of 0.0 to 30 and 60 μM, respectively. In the presence of SC-79, the corresponding Annexin V percentages were suppressed from 11.07% ± 0.20% to 13.94% ± 0.25% and 18.89% ± 0.29%, respectively. Upon addition of the combination of SC-79 with MK-2206, the Annexin V percentages were correspondingly restored to 14.31% ± 0.38%, 22.09% ± 0.33%, and 38.99% ± 0.36% (Fig. 7c). Together with the evidence showing high downregulation of the AR, PI3K, and Akt (Figs. 5a, 7), Bic acted via non-genomic signaling and also an anti-survival pathway. As is well known, the PI3K-Akt pathway acts as a survival signal, promoting mesangial cell survival, and inhibits apoptosis *in vivo* via NF-κB and Bad^49^.

It is worth noting that the PI3K/Akt pathway is composed of a variety of bifurcating and relevant kinase cascades^50^. Thus, understanding this pathway would provide a variety of potential targets for clinical therapy.

### Increased migratory activity was enhanced by Bic therapy

As is well cited, renal mesangial cells and fibroblasts are crucially involved in many progressive renal diseases^51^. Mesangial cell migration through the mesangial matrix and into the pericapillary space in response to PDGF is a feature of a number of renal diseases^52,53^. PDGF signaling triggers stromal recruitment and may be involved in the epithelial-to-mesenchymal transition (EMT), thereby affecting fibrogenesis and metastasis^54^.

**Figure 8:**
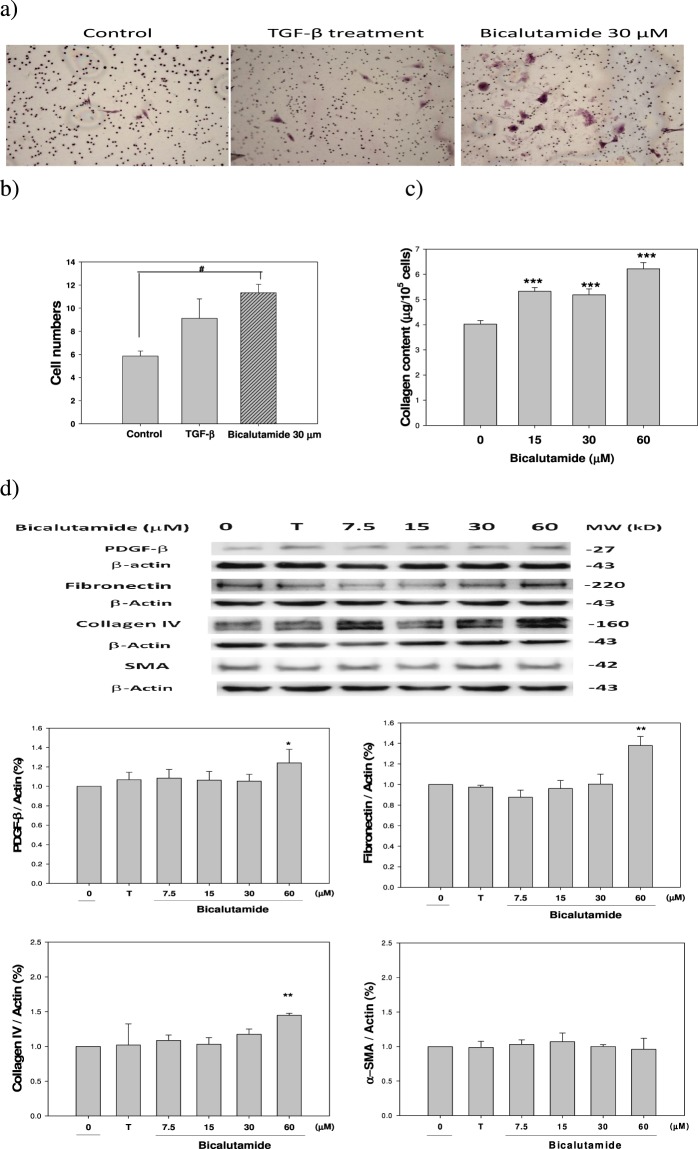
Effect of bicalutamide and TGF-β on the collagen synthesis and associated fibrogenic proteins in RMC cells. (**a**) Transwell assay staining of the control, TGB-β-treated, and bicalutamide-treated RMC cells. (**b**) Quantification of the cell numbers on the transwell assay. (**c**) Quantification of sirius red for the collagen synthesis (numeral data was shown as Supplementary Table 1); and (**d**) Western blot on the expression of fibrogenic proteins PDGF-β, fibronectin, collagen IV, and SMA. Experiments were performed in triplicate and statistically treated (n = 3). The dose of bicalutamide used was 7.5, 15, 30 and 60 μM, respectively. T means Testosterone 100 nM.

We found that Bic did not show a proliferative effect but only increased cell migration (Fig. 8a,b), a result inconsistent with those of Boor *et al*.^51^ and Andrae *et al*.^54^. Results implied that the mechanism of action regarding PDGF expression was biased by Bic therapy. At this point, we also examined the total collagen content and found that the collagen content (per 10^5^ cells) was stimulated by Bic in a dose-dependent manner, increasing from 4.02 ± 0.15 (control) to 5.32 ± 0.15, 5.19 ± 0.23, and 6.22 ± 0.25 μg (*p* < 0.001) at Bic doses of 15, 30, and 60 μM, respectively (Fig. 8c, Supplementary Table S1).

By following the pioneering hypothesis of Arnoni *et al*.^55^ with slight modification, RF may be initiated by Bic in the initial stage via upregulating PDGFs; hence cell proliferation was completely halted (Fig. 1e), however the upregulation of PDGF-β was only noted significantly when Bic at doses of 60 μM (p < 0.05) in our results (Fig. 8d). Literature has demonstrated that glomerular cell apoptosis is associated with a decrease in glomerular cells and the accumulation of extracellular matrix (ECM) during the progression of glomerulosclerosis^56^. The ECM can influence survival and apoptosis of several cell lineages, including glomerular mesangial cells^56^. In the middle stage, the upregulated total collagen, fibronectin and collagen IV synergistically induced myofibroblast transdifferentiation (MFT), leading to RF^56^. These expressions were apparently noted in our results. Total collagen (Fig. 8c), fibronectin and collagen IV (Fig. 8d) were much more highly upregulated at 60 μM Bic, as contrast, SMA was totally unaffected (Fig. 8d), underlying the possible role of high Bic in inducing RF. Taking together, Bic at low experimental doses (15 and 30 μM) failed to initiate the key triggering PDGF signaling (Fig. 8d), but apparently had affected the total collagen (Fig. 8c). When further applying higher dose of testosterone (500 nM) to the Bic (60 μM)-treated cells, only a slight yet insignificant degree of attenuation of fibronectin was observed (data not shown). The reasons could be that (1) Bic prominently suppressed the overall consumption rate of coexisting testosterone (Fig. 2), thus the effect of testosterone was greatly inhibited in co-therapy; (2) The apoptotic and fibrogenic pathways are inherently two separate and totally different pathways; and more relevantly, (3) Downregulation of antifibrotic signaling proteins needs to be expressed in parallel with upregulation of fibrogenic signaling proteins. This observation led to the speculation that cell fibrosis and apoptosis in this cell model might require a much longer duration than was ever implied; thus, the fibrotic phenomenon is indeed extremely hard to replicate in a cell model. A recently published clinical study indicated that correlating acute kidney injury (AKI) with chronic kidney disease (CKD) challenges the conventional pathological concept of focusing on tubular epithelial cells and the basis that epithelial cells can control and regulate fibroblast phenotype^57^, while our RMC model in fact revealed a linking of AKI with further possible RF, which needs to be examined in future extensive research.

In reality, fibrosis occurring in different organs is contributed to by various sources and different phenotypes of myofibroblasts^58^. Consequently, a better knowledge of the molecular mechanisms associated with the appearance of differentiated myofibroblasts in each pathological status could provide much help in understanding fibrogenic development and more importantly, in planning strategies aimed at its prevention and therapy.

### The extrinsic pathway upregulated by Bic is attenuated by co-treatment with testosterone

**Figure 9:**
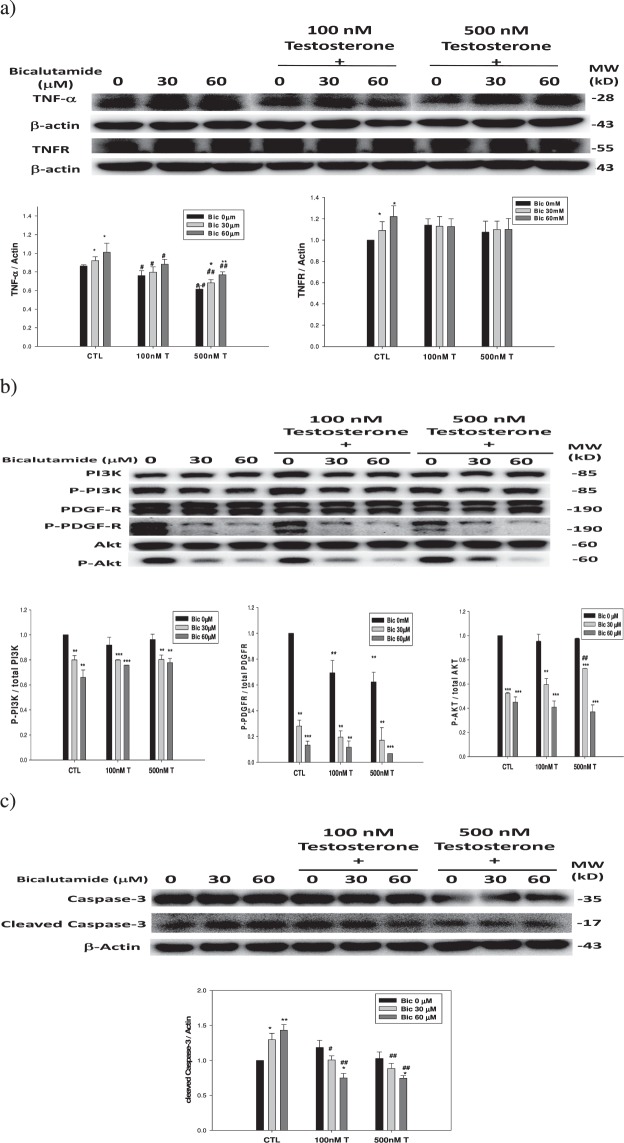
Dose dependent effect of co-treatment with testosterone and bicalutamide on the survival and apoptotic pathways in the RMC cells. (**a**) TNF-α and TNFR. (**b**) Total and phosphorated PI3K, PDGF-R, and Akt. (**c**) Caspase-3 and cleaved caspase-3. Experiment was performed in triplicate and statistically treated (n = 3). The symbol ‘*’ compares within the same group; and ‘#’ compares among groups. *p < 0.05, **p < 0.01, ***p < 0.001. ^#^*p* < 0.05, ^##^*p* < 0.01, ^###^*p* < 0.001.

As mentioned, Bic was able to inhibit both the cytosolic and nuclear AR (Fig. 5); so, we further examined whether testosterone could competitively and efficiently attenuate such a phenomenon and associated signaling pathways. As we found, TNF-α (Fig. 9a) and cleaved caspase-3 (Fig. 9c) were all ameliorated by co-treatment with testosterone at a dose of ≥100 nM, and it seemed that TNFR and p-PI3K were not significantly affected by testosterone (Fig. 9). In contrast, p-Akt (Fig. 9b) was prominently attenuated at a testosterone dose of ≥500 nM.

To summarize, Bic has been widely manipulated in ADT; however, its adverse effects have also been emerging. In the RMC model, Bic was found to upregulate the extrinsic TNF-α → NF-κB → caspase-3 death pathway, and PDGF-fibronectin-collagen IV fibrogenic pathways, and downregulate PI3K-Akt survival, thereby impacting the prognosis of RF. Co-treatment with testosterone showed a prominent suppressive effect, suggesting that an intervention with testosterone could clinically attenuate such adverse effects. However, since Bic can inhibit membrane transport and consumption rates of testosterone, a higher dose of testosterone is recommended.

## Conclusions

Bic may provoke renal mesangial damage and possibly, ultimately fibrosis, *via* multiple mechanisms including androgen deprivation, upregulating the extrinsic TNF-α → NF-κB → caspase-3 death pathway, and downregulating the PI3K-Akt survival pathway and PDGF-fibronectin-collagen IV fibrogenic pathway. Thus, long-term Bic manipulation as an antiandrogenic therapy for treating PCa may otherwise elicit certain adverse effects. Clinically, several strategies can be suggested with respect to prescriptions of antiandrogens like Bic, e.g., therapy modifications to reduce the adverse side effects of Bic by co-manipulation with an appropriate dose of testosterone.

In conclusion, this cell model reveals that Bic can induce renal mesangial damage via multiple mechanisms. As Bic can suppress the transport and intracellular consumption rates of coexisting testosterone, an appropriate dose and application of testosterone should be carefully considered.

## Supplementary information

All supplementary information

## Data Availability

The datasets generated and analyzed during the current study are available from the corresponding author upon reasonable request.
