# Supplementary material for: Renal Damaging Effect Elicited by Bicalutamide Therapy Uncovered Multiple Action Mechanisms As Evidenced by the Cell Model
Source: Sci Rep. 2019 Mar 4;9:3392. doi: 10.1038/s41598-019-39533-3 (PMC6399217; doi:10.1038/s41598-019-39533-3)

Renal Damaging Effect Elicited by Bicalutamide Therapy Uncovered Multiple Action Mechanisms As Evidenced by the Cell Model

Chiung Chi Peng[1], Chang-Yu Chen[2], Chang-Rong Chen[3], Chang-Jui Chen[2], Kun-Hung Shen[4,5], Kuan-Chou Chen[1,6,7,*], Robert Y. Peng[8]

[*]Corresponding author: Kuan-Chou Chen, kuanchou@tmu.edu.tw

Supplement

Fig. S1. Analysis of Annexin V/7-ADD flowcytometry. RMC cells were treated with (a) bicalutamide, (b) bicalutamide plus SC79 and (c) bicalutamide plus SC79 combined with MK2206.

Fig. S2. The original blot for Fig. 9a. Protein expression of TNFα signaling.

Table S1. Collagen content in the RMC cells induced by different doses of bicalutamide.

Table S2. The NAR Labs report on the diabetic nephropathy from the animal pathological examination.

Table S2-a) Histopathology incidence with scoring.

Table S2-b) figure. The cluster plot of the pathological scoring.

Based on the scoring from Table S2-a, the scores indicating the pathological severity among the four groups were re-plotted in Table S2-b figure as cluster diagrams. As seen although bicalutamide (Bic) did not elicit any influence in the normal control (CTL) +Bic, in DM subjects Bic apparently has shown severer pathological changes compared to the DM group. Hence we performed cell model experiment with RMC cell line cultured in high glucose medium to mimic such a DM condition.

(CTL: control, CTL+Bic: control and bicalutamide, Bic: bicalutamide, DM+Bic: diabetes and bicalutamide.)

Fig. S1.

(a)

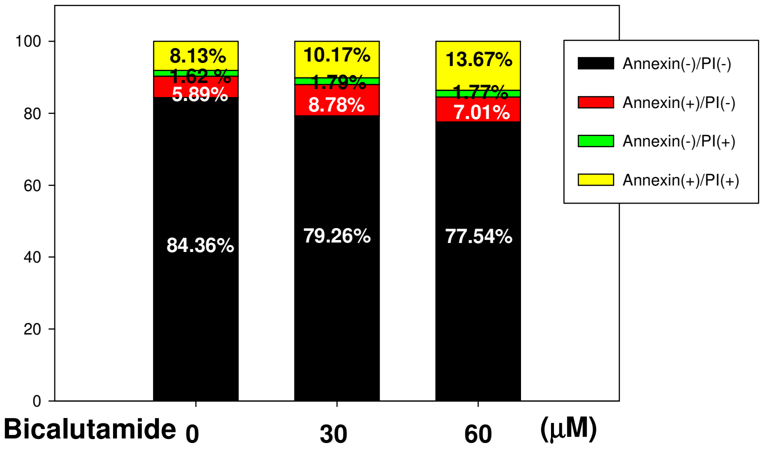

100
80
60
40
20
0
8.13%
1.62 %
5.89%
84.36%
10.17%
1.79%
8.78%
79.26%
13.67%
1.77%
7.01%
77.54%
Annexin(-)/PI(-)
Annexin(+)/PI(-)
Annexin(-)/PI(+)
Annexin(+)/PI(+)
Bicalutamide 0
30
60
(μM)

(b)

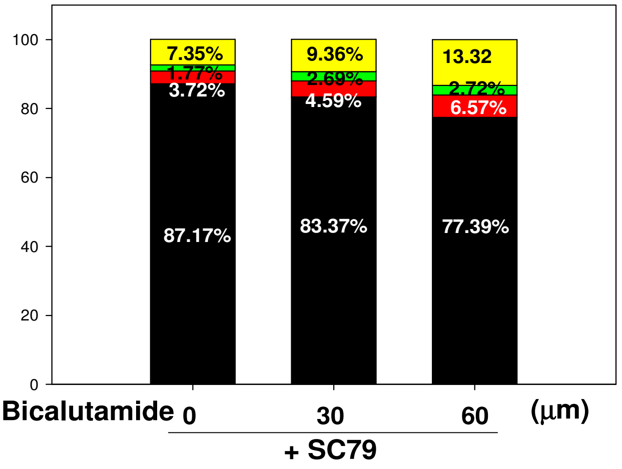

100
80
60
40
20
0
7.35%
1.77%
3.72%
87.17%
9.36%
2.69%
4.59%
83.37%
13.32
2.72%
6.57%
77.39%
Bicalutamide 0
30
60
(μm)
+ SC79

(c)

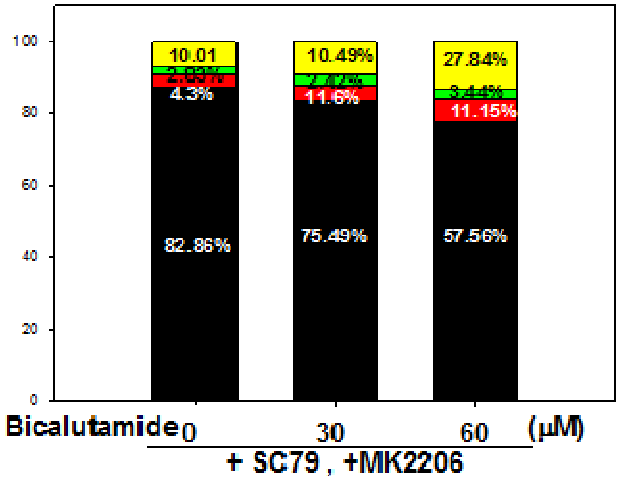

100
80
60
40
20
0
10.01
4.3%
82.86%
10.49%
11.6%
75.49%
27.84%
11.15%
57.56%
Bicalutamide 0
30
60
(μM)
+ SC79 , +MK2206

Fig. S2.

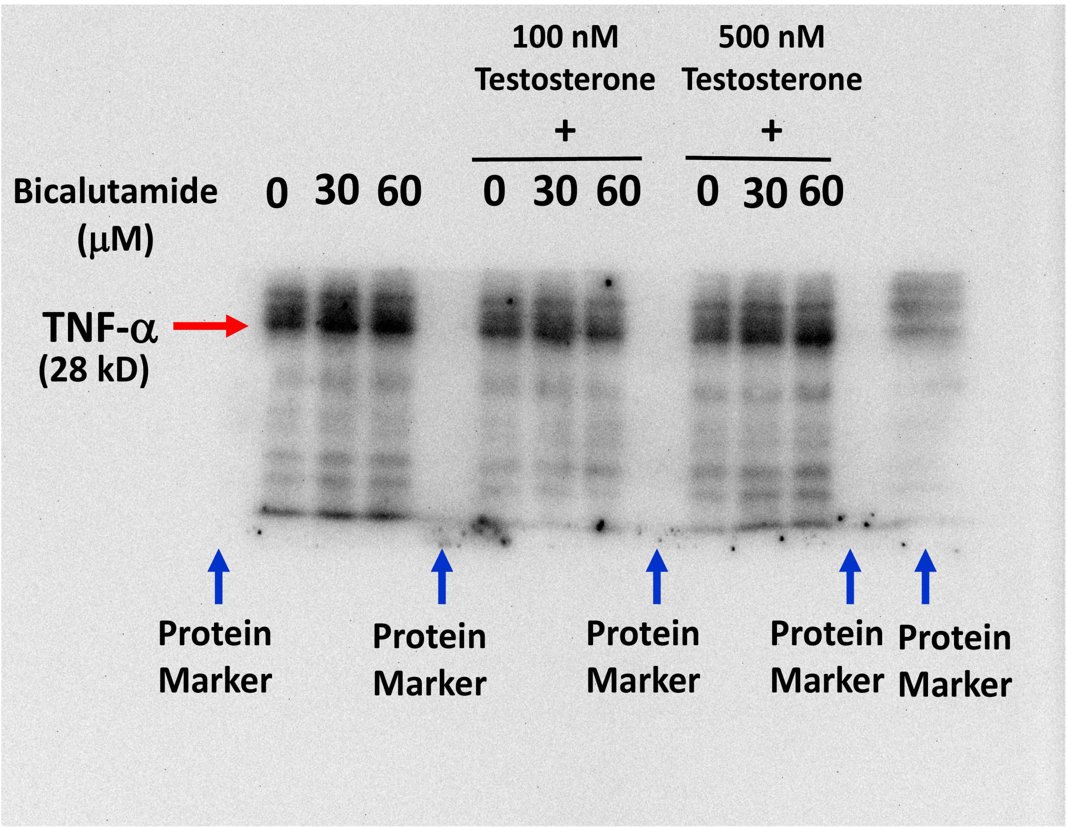
100 nM
Testosterone
+
500 nM
Testosterone
+
Bicalutamide
(μM)
0 30 60
0 30 60
0 30 60
TNF-α
(28 kD)
Protein
Marker
Protein
Marker
Protein
Marker
Protein
Marker
Protein
Marker

Supplement Tables

Table S1. Collagen content in the RMC cells induced by different doses of bicalutamide.

**Bicalutamide**	**Collagen**[#] **(μg/well)**	**Cell number (×10⁵/well)**	**Collagen content (μg/10⁵ cell)**
0 μM	21.54±0.78	5.36±0.88	4.02±0.15
15 μM	20.10±0.57*	3.78±0.27***	5.32±0.15***
30 μM	16.10±0.71***	3.10±0.76***	5.19±0.23***
60 μM	9.98±0.40***	1.61±0.66***	6.22±0.25**

[#]Collagen (μg/well) = [OD_{540}-(OD_{605} × 0.291)]/37.8× 1000 (Catalog # 9046 Chondrex, Inc.).

*p<0.05; ** p <0.01; ***p<0.001.

Table S2. The NAR Labs report on the diabetic nephropathy from the animal pathological examination.

Table S2-a) Histopathology incidence with scoring

**Group**	**Control**					
**Animal ID**	**1-4**	**5-1**	**5-2**	**5-3**	**5-4**	**5-5**
**Kidney**				X		
Diabetic nephropathy						
Hyaline cast, tubule	1	1	1		1	
Inflammatory cell infiltration, pelvis		1				1
Mineralization, tubule	1					

**Group**	**Control + Bicalutamide**						
**Animal ID**	**1-1**	**1-3**	**6-1**	**6-2**	**6-3**	**6-4**	**6-5**
**Kidney**					X		
Diabetic nephropathy							
Hyaline cast, tubule	1	1				1	1
Inflammatory cell infiltration, pelvis				1		2	1
Mineralization, tubule			1				1

**Group**	**Diabetes mellitus (DM)**						
**Animal ID**	**3-4**	**3-5**	**4-1**	**4-2**	**4-3**	**4-4**	**4-5**
**Kidney**							
Diabetic nephropathy	2	2	2	2	2	2	3
[illegible]	1	1			1		
Inflammatory cell infiltration, pelvis	1	1			2	2	1
Mineralization, tubule							

**Group**	**Diabetes mellitus + Bicalutamide**						
**Animal ID**	**2-2**	**2-3**	**2-4**	**2-5**	**3-1**	**3-2**	**3-3**
**Kidney**							
Diabetic nephropathy	2	3	3	3	1	3	1
Hyaline cast, tubule	1						1
Inflammatory cell infiltration, pelvis					1		1
Mineralization, tubule							

The pathological examination was conducted by Dr. Chen T.Y. and Lee K.H. in Diagnostic Laboratory of Laboratory Rodents, National Laboratory Animal Center (NLAC, Taipei, Taiwan). The HE tissue staining and the classification of the degree of lesions were carried out as follows:

1.The required tissues of animals were embedded in paraffin, sectioned at 3-5 μm in thickness, and stained with hematoxylin and eosin (H&E). X=Not remarkable lesion

2. A histopathological evaluation was performed on the submitted tissues/organs of all test animals. Severity of toxicity lesions was graded according to the methods described by Shackelford et al. (Toxicologic Pathology, Vol 30, No 1, pp93-96, 2002). Degrees of lesions were graded histopathologically from one to five depending on severity (1 = minimal (< 1%); 2 = slight (1–25%); 3 = moderate (26–50%); 4 =

moderately severe (51–75%); 5 = severe/high (76–100%).

Table S2-b) figure. The cluster plot of the pathological scoring.

Based on the scoring from Table S2-a, the scores indicating the pathological severity among the four groups were re-plotted in Table S2-b figure as cluster diagrams. As seen although bicalutamide (Bic) did not elicit any influence in the normal control (CTL) +Bic, in DM subjects Bic apparently has shown severer pathological changes compared to the DM group. Hence we performed cell model experiment with RMC cell line cultured in high glucose medium to mimic such a DM condition.

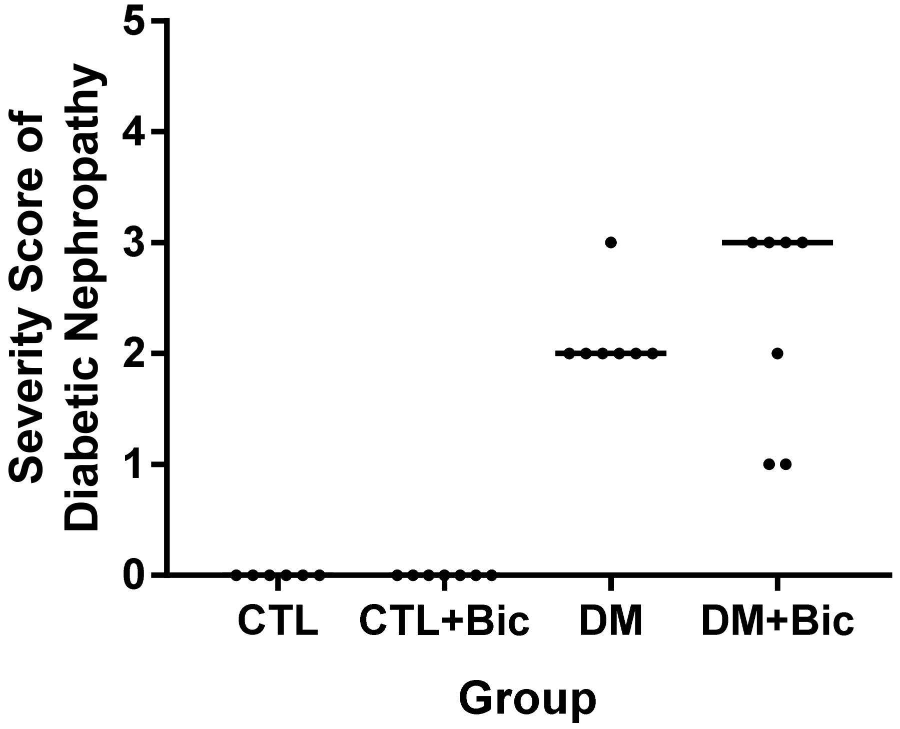

Supplement: Supplementary file 1 — All supplementary information [file 41598_2019_39533_MOESM1_ESM.pdf]
